# Genetic mutations in primary and metastatic tumors of a rare mixed neuroendocrine carcinoma and high-grade serous ovarian cancer

**DOI:** 10.1007/s00795-025-00453-z

**Published:** 2026-02-07

**Authors:** Qiqi Wang, Fenghui Zhao, Shoufeng Chang, Fenglei Liu, Wei Cai, Yamei Dang

**Affiliations:** Gansu Provincial Hospital, Lanzhou, 730000 China

**Keywords:** Epithelial ovarian cancers, Neuroendocrine tumor, Mixed ovarian tumor, Metastasis, Next generation sequencing

## Abstract

Neuroendocrine carcinoma and high-grade serous ovarian cancer (HGSCO) form a rare mixed ovarian tumor. Ovarian cancer is the most lethal gynecological malignancy, with HGSCO being the most common (60–70%) and aggressive subtype, characterized by insidious symptoms and poor prognosis. Neuroendocrine carcinoma (NEC) is a poorly differentiated, high-grade tumor. Their coexistence challenges individualized therapy and confers a poor prognosis. In this case, both components were present in the ovaries, with lung NEC nodules detected 2 years later. Second-generation sequencing revealed shared TP53, PRDM1, and KIT mutations in all three tumors, indicating a common origin from a pluripotent cancer stem cell and suggesting lung NEC metastasized from the ovary. A SPEN mutation was unique to HGSCO, implying a role in HGSCO differentiation, while a PLCG2 mutation occurred only in metastatic lung NEC, indicating a potential key role in lung metastasis.

## Introduction

Mixed neuroendocrine–non-neuroendocrine neoplasms (MiNENs) are mixed epithelial tumors composed of neuroendocrine and non-neuroendocrine tumors. Each component was an independent tumor identifiable by morphology and immunohistochemistry, and the proportion of each component was ≥ 30%. In most MiNENs, both tumor components are poorly differentiated, with organ-specific cancers mixed with NEC [1].

It can occur in various organs throughout the body, with a rising incidence. The malignant degree of MiNENs is higher. The presence of two tumor components at the same time poses a major challenge for individualized tumor therapy, and the prognosis is generally poor [2, 3].

Ovarian cancer represents the most lethal gynecological malignancy. The incidence of ovarian cancer accounts for about 4% of the total malignant tumors in women, ranking the third. It is projected that there will be approximately 19,680 new ovarian cancer cases and 12,740 ovarian cancer deaths worldwide by 2024 [4]. According to the epidemiological analysis of malignant tumors in China in 2016, the incidence rate of ovarian cancer was 8.47 per 100,000, and the mortality rate was 4.04/100,000 [5]. High-grade serous ovarian cancer (HGSCO) is the most common and aggressive histotype of ovarian cancer (60–70%), with insidious symptoms and the worst prognosis. Due to its hidden onset and high degree of malignancy, it leads to a high fatality rate and poses a serious threat to women’s lives [6]. The etiology is not clear, and may be related to many factors such as heredity, fertility and reproductive endocrinology [7]. Neuroendocrine carcinoma (NEC) is a kind of neuroendocrine tumor with poor differentiation and high malignancy, which can originate from all parts of the body [8, 9]. Occurrence of neuroendocrine carcinomas (NECs) in ovary has rarely been reported [10].

There are few studies on the pathogenesis of mixed cancer in ovary. Are the various tumor components of mixed cancer in the same patient caused by different driver gene mutations? Interestingly, both HGSCO and NEC components were present in the ovaries in this case, and the nodules of NEC were found in the lungs 2 years later. Therefore, we are the first to speculate whether the nodules of lung cancer are due to metastasis of the NEC component of ovarian mixed carcinoma or the lung primary. If it was the NEC of ovarian that had metastasized to the lung, why did the NEC component metastasize in this case, but not in HGSCO? Are there key genes that play an important role in the metastasis of NEC in this case?

In order to answer the above questions, we sequenced the components of HGSC and NEC respectively by next-generation sequencing, and compared the differences of gene mutations between them. If there is a different gene mutation, does that gene mutation cause different tumors to develop at the same site? At the same time, next generation sequencing of lung neuroendocrine carcinoma was also performed to compare the differences in genetic mutations between ovarian and lung neuroendocrine carcinoma. Next generation sequencing analysis was conducted to find out whether there were common mutations and determine whether NEC was the same tumor origin, that is, the pulmonary neuroendocrine carcinoma was caused by the metastasis of the ovarian neuroendocrine carcinoma.

## Methods

### Immunohistochemistry

The specimens were fixed with 3.7% neutral formaldehyde, embedded in paraffin, dewaxed by routine method. The specimens were successively sliced 4 μm thick and stained by HE. Immunohistochemistry was performed by EnVision two-step method with diaminobenzidine (DAB) for color development. The primary antibodies were PAX-8, WT1, P53, Syn, CD56. The antibody and EnVision kit were purchased from Fuzhou Maixim Biotechnology Development Co., LTD., and operated according to the instructions.

### Next generation sequencing and analysis

Tumor enrichment experiment: The paraffin tissue to be measured was cut into 5 μm thick sections and collected on slides. Delineate the tumor cell area under an optical microscope. Non-target tumor tissue was removed with disposable blade and the remaining tumor tissue was collected. Tissue DNA was extracted with FFPE DNA Kit (OMEGA); After DNA quality testing and in accordance with Genecast Comprehensive (Genecast) product instructions, sequencing was performed by Illumina Nextseq 550Dx platform to analyze gene point mutations in all exon regions of 769 genes.

## Results

### The patient’s ovarian tumors were diagnosed as mixed neuroendocrine carcinoma and high-grade serous ovarian cancer

**Fig. 1:**
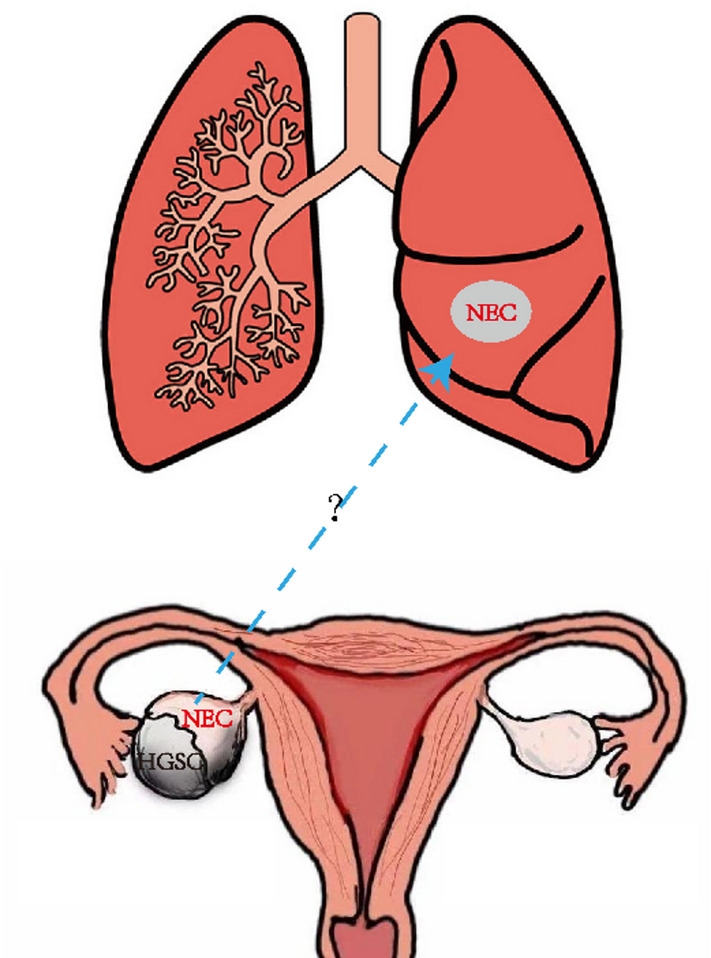
The model of ovarian primary tumor and lung tumor in this case. Two components of high-grade serous ovarian cancer and neuroendocrine carcinoma were found in the ovarian tumor, and neuroendocrine carcinoma nodules were found in the lung

**Fig. 2:**
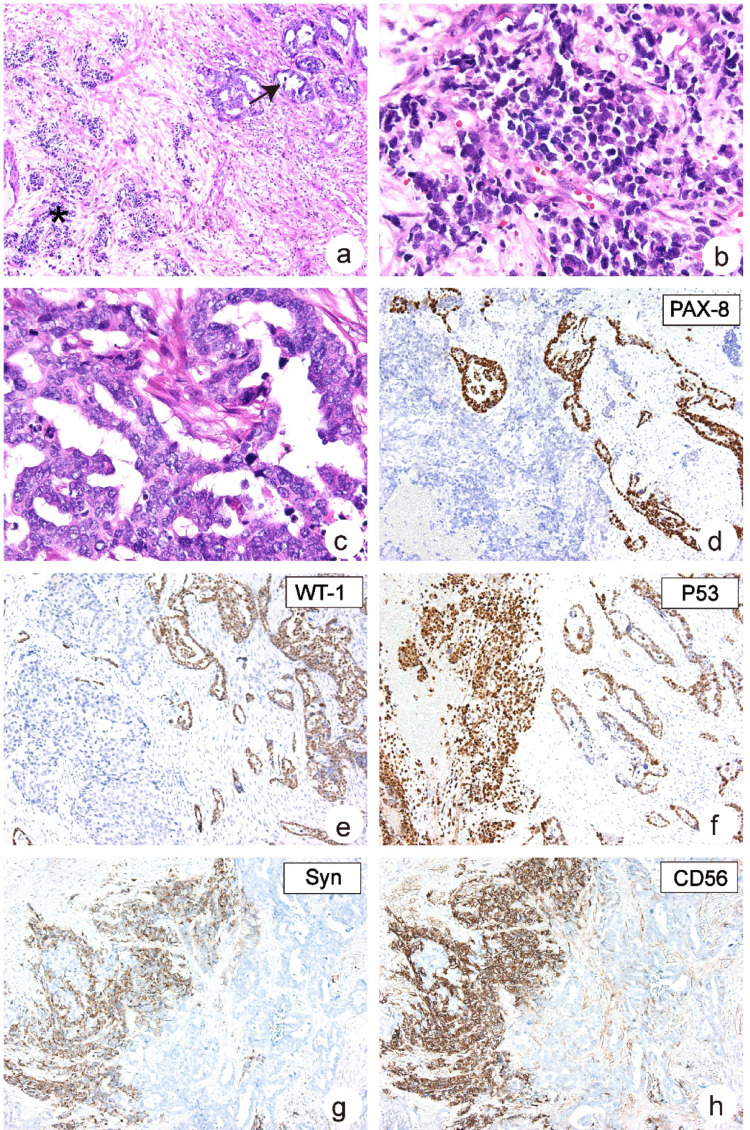
Histopathological features of MiNENs of ovary (NEC and HGSCO). **a** Low-magnification images from MiNENs consisting of NEC (asterisk) and HGSCO (arrow) (Hematoxylin-and-eosin, H&E × 40); **b** The images contain areas of NEC of MiNENs (H&E × 400); **c** The images contain areas of HGSCO of MiNENs (H&E × 400); IHC for PAX-8 (**d**) and WT1 (**e**) staining positively in the HGSCO and negatively in the NEC regions (H&E × 100); **f** IHC for P53 staining positively in both the HGSCO and NEC regions (H&E × 100); IHC for Syn (**g**) and CD56 (**h**) staining positively in the NEC and negatively in the HGSCO regions (H&E × 100)

In this case, a 59-year-old female patient was found with a nodular mass with a size of 7 × 5 × 3 cm in the right ovary. The cross-section of the tumor is gray and white. Two groups of tumor cells with obvious morphological differences were observed under optical microscope (Figs. 1 and 2a, b), and the proportion of cells in each group was more than 30%. A group of cells with immunohistochemical staining positive for PAX-8 (Fig. 2d), WT-1 (Fig. 2e), and P53 (Fig. 2f) combined with cell morphology were diagnosed as high-grade serous ovarian carcinoma. Another group of cells with immunohistochemical staining positive for Syn (Fig. 2g) and CD56 (Fig. 2h) combined with cell morphology were diagnosed as neuroendocrine carcinoma. Finally, combined with the results of HE staining and immunohistochemical staining, the ovarian tumor of the patient was diagnosed as mixed neuroendocrine carcinoma (NEC) and high-grade serous ovarian cancer (HGSCO).

### The patient’s pulmonary nodules were diagnosed as neuroendocrine carcinoma, which was highly considered to be metastatic

**Fig. 3:**
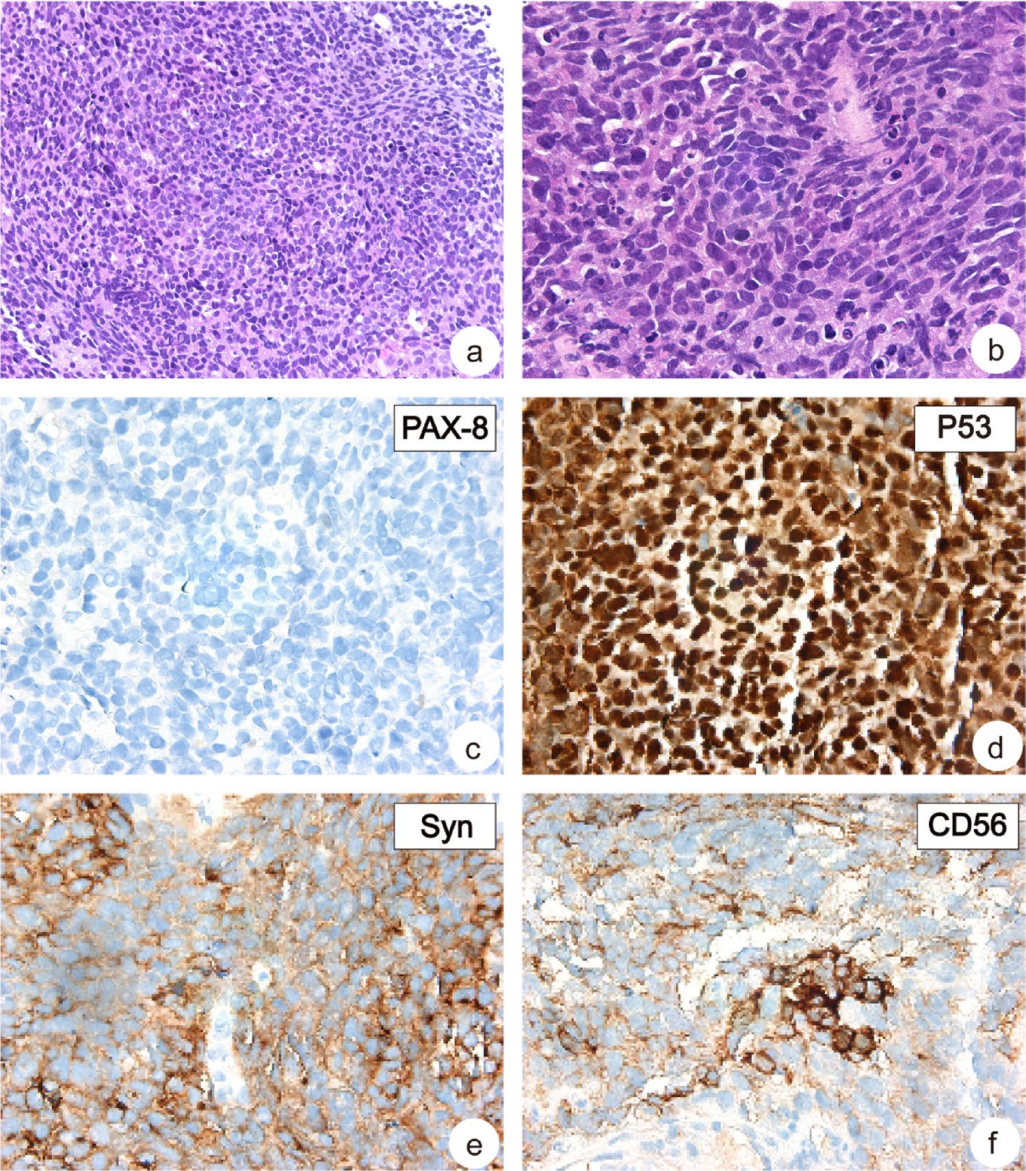
Histopathological features of NEC of lung. **a** Low-magnification image from NEC of lung (H&E × 200); **b** High-magnification images from NEC of lung. (H&E × 400); **c** IHC for PAX-8 staining negatively in the NEC of lung (H&E × 400); IHC for P53 (**d**), Syn (**e**) and CD56 (**f**) staining positively in the NEC of lung (H&E × 400)

After treatment, nodules were found in the lung 2 years later (Fig. 1). Small, round tumor cells of uniform size were observed under the light microscope in lung puncture tissue biopsy, morphologically very similar to the neuroendocrine carcinoma components in ovarian mixed carcinoma (Fig. 3a, b). Immunohistochemical staining was positive for P53 (Fig. 3d), Syn (Fig. 3e), CD56 (Fig. 3f), and negative for PAX-8 (Fig. 3c). Combined with cell morphology, the diagnosis was neuroendocrine carcinoma, highly considering metastasis. To investigate whether lung neuroendocrine carcinoma is derived from ovarian neuroendocrine carcinoma, we performed next generation sequencing. If the lung and the ovarian neuroendocrine carcinoma share a common point mutation, it is demonstrated that the lung neuroendocrine carcinoma is a metastatic ovarian neuroendocrine carcinoma.

### Next generation sequencing suggested that pulmonary neuroendocrine carcinoma was metastatic from ovarian neuroendocrine carcinoma

**Fig. 4:**
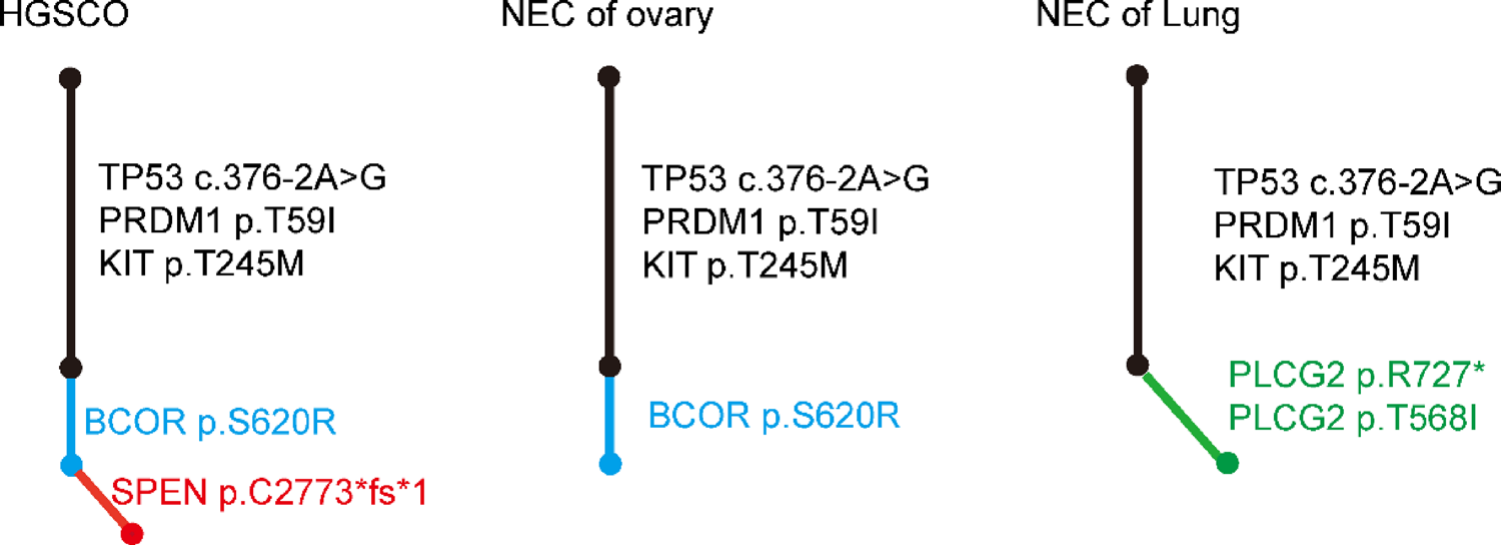
Phylogenetic trees constructed based on the point mutations of HGSCO, NEC of ovary, and NEC of Lung. For each case, the trunk (black) represents variants common to both components, while the branches (blue, green, red) represent variants exclusive to each respective component. Trunk/branch lengths are proportional to the number of point mutations, as indicated next to each segment

The next generation sequencing results showed that HGSCO had five point mutations: *TP53* c.376-2A > G, *PRDM1* p.T59I, *KIT* p.T245M, *BCO*R p.S620R, *SPEN* p.C2773*fs*1; The NEC of ovary had four point mutations: *TP53* c.376-2A > G, *PRDM1* p.T59I, *KIT* p.T245M, *BCOR* p.S620R; The NEC of lung had five point mutations: *TP53* c.376-2A > G, *PRDM1* p.T59I, *KIT* p.T245M, *PLCG2* p.R727*, *PLCG2* p.T568I (Fig. 4). All three tumors contain three mutations: *TP53* c.376-2A > G, *PRDM1* p.T59I, *KIT* p.T245M. This indicates that the three tumors are derived from a pluripotent cancer stem cell, illustrating the evolutionary relationships. It is suggested that pulmonary neuroendocrine carcinoma was metastatic from ovarian neuroendocrine carcinoma. However, the *BCOR* p. S620R mutation was present in NEC of ovary but absent in NEC of lung. The reason for this may be the spatial heterogeneity of the tumors. The *SPEN* p.C2773*fs*1 mutation is a differential gene for ovarian neuroendocrine carcinoma and ovarian serous carcinoma.

### SPEN may be the key gene that causes pluripotent cancer stem cells to differentiate into high-grade serous ovarian carcinoma

In this case, two tumors were found in the ovary of the patient, and whether there was a specific mutation that led to the formation of two different tumors. After the next generation sequencing analysis, it was found that: the *SPEN* p.C2773*fs*1 mutation is a differential gene for ovarian neuroendocrine carcinoma and ovarian serous carcinoma.

SHARP showing features for dna binding is a potent transcriptional repressor whose repression domain (RD) interacts directly with target gene [11, 12]. Therefore, We hypothesized that SPEN may be the key gene that causes pluripotent cancer stem cells to differentiate into high-grade serous ovarian carcinoma. That’s something that we’re going to have to work on in the future, and we’re going to test this hypothesis experimentally.

### PLCG2 may play a key role in the metastasis of ovarian neuroendocrine carcinoma to the lung

By next generation sequencing, we identified *PLCG2* mutations in metastatic lung neuroendocrine carcinomas but not in primary neuroendocrine carcinomas. *PLCG2* encodes phospholipase Cγ2 (PLCγ2), an enzyme with a critical regulatory role in various pathways. The production of the second messenger molecules diacylglycerol (DAG) and inositol 1,4,5-trisphosphate (IP3) is mediated by activated PLCγ2. It is a crucial enzyme in transmembrane signaling [13]. Similarly, we hypothesized that PLCG2 may play an important role in the signaling of tumor cell metastasis. This needs more experimental verification.

## Discussion

In this study, we identified *SPEN* gene, which may lead to different tumor differentiation direction, and *PLCG2* gene (Fig. 4**)**, which may lead to metastasis of neuroendocrine carcinoma, by next generation sequencing of a rare metastatic mixed neuroendocrine carcinoma and high-grade serous carcinoma. Mixed neuroendocrine carcinoma and high-grade serous carcinoma contain significantly different tumor components. The treatment options of the two tumors are completely different, which poses a serious hindrance to the individualized tumor treatment and prognosis of patients [14]. In this study, we compared the gene mutations of two tumors by next generation sequencing, and found that the two tumors shared three point mutations: *TP53* c.376-2A > G, *PRDM1* p.T59I, and *KIT* p.T245M. This indicates that tumorigenesis is a process of accumulation of multiple gene mutations, and each gene mutation will change the tumor [15].

*TP53* gene is a tumor suppressor gene and encodes tumor protein p53 (a transcription factor), which plays an important role in inhibiting the occurrence of cancer by inducing downstream anti-tumor responses such as DNA repair and apoptosis in response to cellular stress (including DNA damage and carcinogenic activation) [16, 17]. *PRDM1* gene is a tumor suppressor gene, encoding protein is PR domain Zinc finger 1 (PRDM1), also known as BLIMP-1 and PRDI-BF1, which is a DNA binding protein containing five zinc finger structures. The *PRDM1* gene has been shown to function by interacting with transcriptional corepressors of the Groucho family, as well as histone modifying factors. The results showed that *PRDM1* gene is an important factor in germ cell lineage, T cell differentiation, homeostasis and cardiac function establishment [18]. *KIT* gene is an oncogene encoding a type 3 transmembrane receptor tyrosine kinase. After the protein encoded by the *KIT* gene is bound to its ligand stem cell factor (SCF), the receptor is activated through dimerization and autophosphorylation. *KIT* gene activation leads to increased intracellular signaling through several pathways, including PI3K, MAPK, and STAT, ultimately leading to cell proliferation and survival [19, 20].

Only *SPEN* mutations differ between ovarian neuroendocrine carcinoma and high-grade serous carcinoma. It is suggested that SPEN plays an important role in the differentiation of pluripotent tumor stem cells. *SPEN* gene is a tumor suppressor gene, encoding a SMRT/HDAC1-related repressor protein (SHARP), which is mainly involved in transcriptional inhibition and embryogenesis and development by regulating Notch, TCF/LEF, and EGFR signaling pathways [21, 22].

Ovarian neuroendocrine carcinoma and lung neuroendocrine carcinoma share three common mutations *ATP53*,* PRDM1*,* and KIT*. This is important evidence for the metastasis of lung neuroendocrine carcinoma from ovarian neuroendocrine carcinoma.

But the *BCOR* mutation is present in ovarian neuroendocrine carcinoma and absent in lung neuroendocrine carcinoma. The reason may be the spatial and temporal heterogeneity of the tumor [23]. It may also be that BCOR does not play a role in the development of neuroendocrine cancer. The *BCOR* gene is a cancer suppressor (BCL6 co-repressor), and the encoded protein is a transcriptional corepressor that can be repressed in a peculiar way when recruited to the promoter region by sequence-specific DNA binding proteins such as BCL6 and MLLT3 [24]. *PLCG2* mutation is present in lung neuroendocrine carcinoma but absent in ovarian neuroendocrine carcinoma. The *PLCG2* gene encodes a protein called phospholipase Cγ2, a calcium-dependent enzyme involved in transmembrane signaling. It can split phospholipids (phosphatidylinositol 4,5-diphosphate (PIP2)) into glycerol diesters (DAG and inositol 1,4,5-triphosphate (IP3) [25]. This suggests that PLCG2 may play an important role in the metastasis of ovarian neuroendocrine carcinoma to the lung.

In the latest WHO classification and some consensus statements, double inactivation of *RB1* and *TP53* has become one of the core molecular features for defining high-grade NEC, used to distinguish it from low-grade neuroendocrine tumors (such as carcinoid tumors, which usually retain *RB1*). Inactivation (mutation or loss) of the *RB1* gene is a hallmark molecular event in high-grade, especially small cell neuroendocrine carcinomas, and has been reported in the vast majority of cases [26].

This case only has *TP53* mutation and no *RB1* mutation or loss, the reason for which may be: ① Many NECs have *RB1* mutations or loss, and *RB1* mutations or loss play an important role in the development and progression of NECs. However, a few cases (such as this case) do not have *RB1* mutations or loss, suggesting that other genes can also lead to the development and progression of NECs, meaning that the NECs in this case may be driven by other genes; ② Limitations in the detection method. We used the target gene capture sequencing method, which only covers hotspot mutation fragments and did not perform whole-exome sequencing. ③The loss of RB1 protein function may not be achieved through gene alteration, but rather through epigenetic silencing (such as hypermethylation of the *RB1* gene promoter) and abnormal post-transcriptional regulation.

There is only one such special specimen in this paper, and the research results are not representative and have no statistical significance. Next, we will continue to collect similar cases for research. However, this paper studies the gene mutations of two tumors and the gene mutations of the primary and metastatic foci in the same individual, which can better explain the polygene theory of tumor development, so as to better carry out individualized tumor treatment.

## Data Availability

The data that support the findings of this case report are available from the corresponding author upon reasonable request.

## Declarations

### Ethnic approval

Ethical approval for this study was obtained from an ethics committee or institutional review board.
